# An observational cohort study on pulmonary function in adult patients with 5q-spinal muscular atrophy under nusinersen therapy

**DOI:** 10.1007/s00415-023-11711-4

**Published:** 2023-04-16

**Authors:** Bogdan Bjelica, Camilla Wohnrade, Alma Osmanovic, Olivia Schreiber-Katz, Susanne Petri

**Affiliations:** 1 Department of Neurology, Hannover Medical School, 1, Carl-Neuberg-Strasse, 30625 Hannover, Germany; 2 Essen Center for Rare Diseases (EZSE), University Hospital Essen, Essen, Germany

**Keywords:** Spinal muscular atrophy, Nusinersen, Pulmonary function, Motor function, Quality of life, Fatigue

## Abstract

**Background:**

Few studies assessed the effect of nusinersen on respiratory function in adult patients with spinal muscular atrophy (SMA). The aim of this single-center study was to analyze pulmonary function and its association with muscle function and quality of life (QoL) in adult patients with 5q-SMA under nusinersen.

**Methods:**

We recorded forced vital capacity (FVC), forced expiratory volume in the first second (FEV1) and peak expiratory flow (PEF) during nusinersen treatment in 38 adult SMA patients. Revised Upper Limb Module (RULM), Hammersmith Functional Motor Scale Expanded (HFMSE), 36-Item Short Form Health Survey (SF-36) questionnaire and Fatigue Severity Scale (FSS) were recorded and correlations between muscle function, QoL, fatigue and respiratory parameters were analyzed.

**Results:**

No differences were detected between mean FVC, FEV1, PEF at different timepoints versus baseline. Ambulatory patients showed significant improvement in mean PEF at month 30, compared to non-ambulatory patients (+ 0.8 ± 0.5 vs. − 0.0 ± 0.5, *p* < 0.05). Patients with fatigue at baseline showed significant improvement in mean PEF at month 10, compared to patients without fatigue at baseline (+ 0.6 ± 0.9 vs. − 0.4 ± 0.5, *p* < 0.05). Physical domains of SF-36 positively correlated with the change in FVC and FEV1. FSS negatively correlated with the change in mean PEF.

**Conclusion:**

Mean pulmonary function remained stable during nusinersen treatment over a period of up to 30 months. Improvement in pulmonary function was associated with improvement in motor function, fatigue and QoL, early after nusinersen initiation.

**Supplementary Information:**

The online version contains supplementary material available at 10.1007/s00415-023-11711-4.

## Introduction

Spinal muscular atrophy (SMA) is a hereditary lower motor neuron disorder characterized by progressive predominantly proximal muscular weakness and atrophy. It represents one of the most common autosomal recessively inherited diseases, with an estimated incidence from 1 in 6000 to 1 in 10,000 live births [1]. SMA is caused by homozygous deletions/mutations in the *survival of motor neuron* (*SMN*) *1* gene located on chromosome 5q13.2, leading to an insufficient production of functional SMN protein. The paralogous *SMN2* gene differs from the *SMN1* gene by a C to T change in the splicing region of exon 7, which ultimately results in reduced splicing efficiency and exclusion of exon 7 [1, 2]. Therefore, *SMN2* gene contributes only slightly to the production of functional SMN protein [1, 2].

SMA can be classified into different groups with a fluent transition based on the age of symptom onset and motor milestones achieved, ranging from SMA type 0 (prenatal manifestation) to SMA type 4 (adult-onset of weakness). Higher *SMN2* copy numbers are associated with reduced disease severity [3].

Respiratory function in SMA is often impaired and dependent on disease severity. The most common cause of mortality in the severe phenotypes of SMA are respiratory complications [4]. A natural history of pulmonary function in SMA patients showed the fastest decline of the forced vital capacity (FVC) during childhood and stabilization during early adulthood in SMA types 1c (age at onset 3–6 months)-3a, while FVC in SMA types 3b and 4 remained stable throughout life [5]. Here, SMA type 3a patients with four *SMN2* copies had a slower longitudinal decline regarding motor and pulmonary function, compared to those with three *SMN2* copies [5]. Trucco et al. found in a large international SMA cohort (*n* = 437) that FVC declined more steeply from 5 to 13 years of age, which was followed by a stabilization after 13 years of age. In this cohort, 39% of patients with SMA type 2 had started non-invasive ventilation (NIV) treatment at a median age of 5 years, while 9% of patients with SMA type 3 required NIV at median age of 15.1 years [6].

In recent years, disease-modifying medications (nusinersen, risdiplam, onasemnogene abeparvovec) have been developed. They can lead to phenotypic stabilization or even improvement and have substantially altered disease prognosis [1]. Nusinersen is an antisense oligonucleotide that affects SMN2 RNA splicing and favors expression of stable and functional SMN protein [1]. While clinical phase 3 trials have only been performed in children up to the age of 12 years [7, 8], more recent studies found that nusinersen effectively improved motor function not only in children but also in adult SMA patients [9, 10]. Few studies have assessed the impact of nusinersen on pulmonary function, so far [11–15]. Chacko et al. reported that nusinersen slowed down the decline in pulmonary function during the first year of treatment in pediatric SMA patients [11]. In adult SMA patients treated with nusinersen, improvement in peak cough flow [12], FVC and forced expiratory volume in the first second (FEV1) [13, 14] and maximal expiratory pressure [15] has been shown. The longest follow-up period of these studies was 14 months, and no study has correlated the change in muscle function, quality of life (QoL) and fatigue with a change in respiratory function in adult SMA patients under nusinersen therapy so far.

The aim of this study was to analyze pulmonary function and its association with muscle function, QoL and fatigue in adult patients with 5q-SMA treated with nusinersen.

## Materials and methods

### Participants

**Fig. 1:**
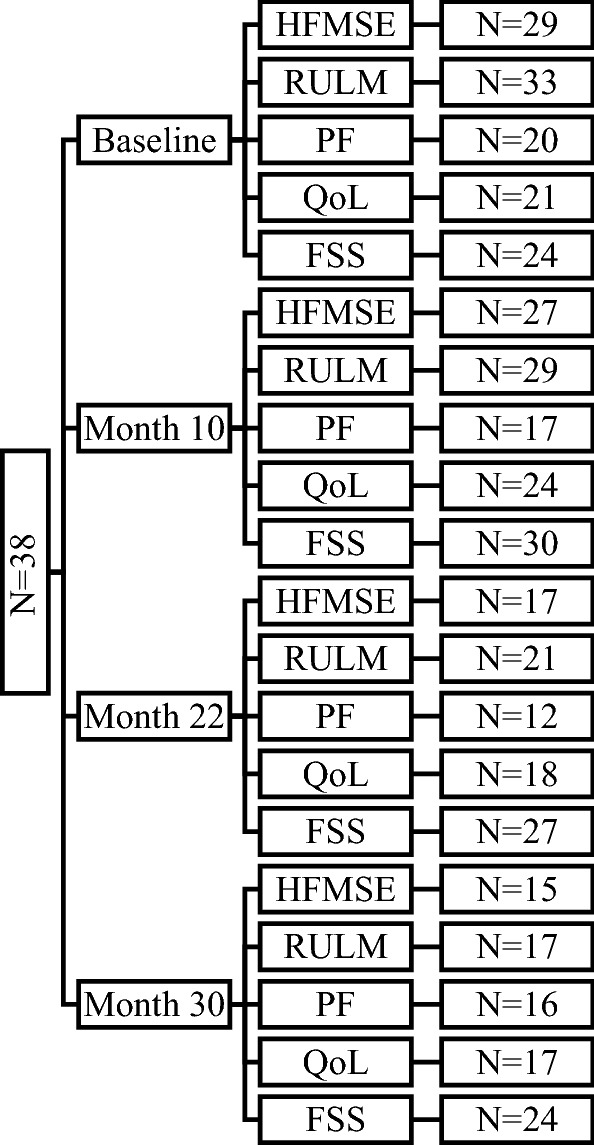
Availability of data (motor function scores, pulmonary function, QoL and fatigue) of included SMA patients at baseline and at follow-up timepoints. *N* number of patients, *HFMSE* Hammersmith Functional Motor Scale Expanded, *RULM* Revised Upper Limb Module, *PF* pulmonary function tests (forced vital capacity (FVC), forced expiratory volume in the first second (FEV1), peak expiratory flow (PEF)), *QoL* quality of life, *FSS* Krupp’s Fatigue Severity Scale

In this prospective, longitudinal, monocentric, observational study, we included all SMA patients who received nusinersen treatment at the Department of Neurology of Hannover Medical School between April 2018 and December 2022. All participants were 18 years or older and had a genetically confirmed diagnosis of 5q-SMA (homozygous deletion of exon 7 or/and exon 8 of *SMN1* gene or compound heterozygous deletion of exon 7 or/and exon 8 of *SMN1* gene together with a point mutation on the second gene copy). Nusinersen was applied intrathecally according to the approved protocol, starting with a loading period (day 0, 14, 28 and 63) followed by administrations every 4 months. The analyzed SMA cohort consisted of 38 patients. Sociodemographic and clinical data including gender, age at therapy start, disease duration, SMA type, *SMN2* gene copy number, ambulatory status (defined as the ability to walk at least 10 m without assistance or use of a device such as cane or a walker [16]), use of wheelchair, presence of scoliosis, use of NIV and presence of percutaneous endoscopic gastrostomy (PEG) were recorded at baseline. Follow-up data were collected at month 10, 22 and 30 of nusinersen treatment. Figure 1 shows the availability of data (motor function scores, pulmonary function scores, QoL and fatigue) of included SMA patients at baseline and during follow-up. Patients’ baseline and follow-up data on respiratory and motor function was lost due to minimum length of hospital stay during COVID-19 pandemic. The data on QoL and fatigue was lost, because the patients refused to fill in the questionnaires or did not fill in the questionnaires properly. The study was approved by the Ethical Board of Hannover Medical School (no. 6269) and all patients gave their written informed consent to participate.

### Assessment of pulmonary and motor function, quality of life and fatigue

Spirometry was performed in sitting or supine position in all patients by a trained and experienced examiner (calibrated device: Ganshorn Medizin Electronic BodyScope^**®**^). FVC, FEV1 and peak expiratory flow (PEF) were registered, and raw scores and percent predicted scores were reported. Predicted values were calculated according to European Community for Steel and Coal [17]. Three attempts were recorded (in liters), and the best test result was used for further analysis. The best result was considered the result with the best PEF. If the recorded values of three attempts differed more than 10% between each other, the spirometry was repeated.

Motor function was assessed by trained professional physiotherapists using the Revised Upper Limb Module (RULM) score [18] and the Hammersmith Functional Motor Scale Expanded (HFMSE) score [19]. The RULM is a disease-specific scale that measures motor function of upper extremities and performance in activities of daily living. It has 20 items, and the patients can score a maximum of 37 points, where higher scores represent better function of upper limbs. The HFMSE is a 33-item disease-specific scale that measures gross motor function, where the patients can score a maximum of 66 points (higher scores representing a better motor function).

The presence and severity of fatigue was assessed using the Krupp’s Fatigue Severity Scale (FSS) [20]. It is a nine-item self-reported questionnaire that measures the severity of fatigue in the time frame of the “past week”, where higher scores represent more severe fatigue. The presence of fatigue was considered if the total score was equal or greater than 36.

The German version of the SF-36 questionnaire [21] was used to assess patients’ health-related QoL. It represents a generic measure that combines eight general health concepts: physical functioning (PF), role physical (RP), bodily pain (BP), general health (GH), vitality (VT), social functioning (SF), role emotional (RE), and mental health (MH). Two main scores that are summarizing these eight general health concepts are the physical composite score (PCS) and mental composite score (MCS), besides the total SF-36 score. The standardized sum-scores of each concept range from 0 to 100, whereby higher values represent better QoL.

### Statistical analysis

We performed the statistical analysis using IBM^®^ Statistical Software Package of Social Science (SPSS^®^, Chicago, IL, USA) version 28. Normality of data was determined using Shapiro–Wilk and Kolmogorov–Smirnov test. Considering the small number of included participants we used non-parametric statistics. The differences between baseline and examined timepoints (month 10, 22 and 30 of nusinersen treatment) were examined using Wilcoxon rank sum test and Fisher’s exact test. Correlations were determined with Spearman’s rank (correlation) coefficient. In the subgroup analysis, we used Mann–Whitney *U* test for independent samples. We included only the patients with SMA type 2 and 3, due to the small number of patients with SMA type 1 and type 4. Level of statistical significance was set at 0.05 and 0.01.

## Results

### Patients’ characteristics

Main sociodemographic and clinical characteristics of enrolled SMA patients at baseline are presented in Table 1. Most of the patients had SMA type 3 (*n* = 21, 55.3%) or SMA type 2 (*n* = 14, 36.8%). Out of all SMA type 3 patients, seven had SMA type 3a and 14 had SMA type 3b. Two patients (5.3%) had SMA type 4, and one patient (2.6%) had SMA type 1. Mean age at the start of nusinersen treatment was 38.4 ± 14.1 years and mean disease duration was 32.5 ± 13.5 years. Almost one third of the analyzed patients were ambulatory (*n* = 11, 28.9%). Twenty-three patients had scoliosis, seven had undergone spinal fusion surgery (spondylodesis), seven were dependent on NIV and two used a PEG. The mean HFMSE score was 24.7 ± 23.6 and the mean RULM score was 22.1 ± 13.2.

**Table 1:** Main sociodemographic and clinical characteristics of all analyzed SMA patients at baseline

SMA features	
*N*	38
Male gender (*n*, %)	24 (61.4)
Age at therapy start (years, mean ± SD)	38.4 ± 14.1
Disease duration (years, mean ± SD)	32.5 ± 13.5
SMA type (*n*, %) SMA type 1 SMA type 2 SMA type 3 SMA type 4	1 (2.6) 14 (36.8) 21 (55.3) 2 (5.3)
*SMN2* copy number < 4 ≥ 4	18 (47.4) 20 (52.6)
Walking ability (n, %) Ambulatory Non-ambulatory	11 (28.9) 27 (71.1)
Wheelchair use (*n*, %) Never Sometimes Always	13 (34.2) 17 (44.7) 8 (21.1)
Scoliosis (*n*, %) **Spondylodesis (*n*, %** out **of all patients with scoliosis)**	23 (60.5) 7 (30.4%)
NIV (*n*, %)	7 (18.4)
PEG (*n*, %)	2 (5.3)
HFMSE score (mean ± SD)	24.7 ± 23.6
RULM score (mean ± SD)	22.1 ± 13.2
FSS score (mean ± SD) % of fatigued	40.1 ± 11.9 66.7%
Total SF-36 score (mean ± SD)	58.6 ± 12.0

*SMA* spinal muscular atrophy, *N* number, *SD* standard deviation, *SMN2* *survival of motor neuron 2* gene, *NIV* non-invasive ventilation, *PEG* percutaneous endoscopic gastrostomy, *HFMSE* Hammersmith Functional Motor Scale Expanded, *RULM* Revised Upper Limb Module, *FSS* Fatigue Severity Scale, *SF-36* The 36-Item Short Form Health Survey

### Pulmonary function and its correlation with motor function during nusinersen treatment

**Fig. 2:**
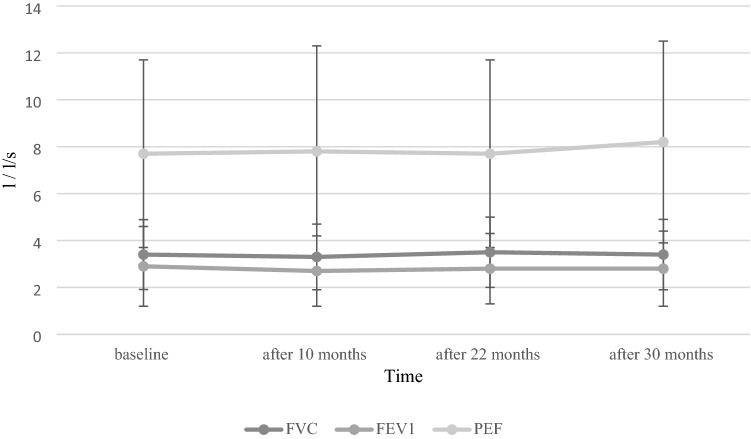
Pulmonary function during nusinersen treatment. *FVC* forced vital capacity, *FEV1* forced expiratory volume in the first second, *PEF* peak expiratory flow, *l* liter, *l/s* liter per second for PEF

Table 2 shows changes in mean FVC, FEV1, PEF, HFMSE, RULM and SF-36 score from baseline to different timepoints during nusinersen treatment. Pulmonary function scores during nusinersen treatment are shown in Fig. 2. No significant differences were detected between mean FVC, FEV1, PEF as well as HFMSE scores obtained at baseline and at different timepoints during nusinersen treatment (*p* > 0.05). Improvement of FVC (difference > 0 between FVC obtained at follow-up vs. baseline) was seen in 50% of patients at month 10 (+ 0.3 ± 0.3; 0.03–0.75), 55% of patients at month 22 (+ 0.2 ± 0.1; 0.03–0.3) and 58% of patients at month 30 (+ 0.2 ± 0.2; 0.03–0.6). Improvement of FEV1 (difference > 0 between FEV1 obtained at follow-up vs. baseline) was seen in 50% of patients at month 10 (+ 0.5 ± 0.5; 0.05–0.1.5), 57% of patients at month 22 (+ 0.3 ± 01.; 0.1–0.5) and 50% of patients at month 30 (+ 0.2 ± 0.1; 0.02–0.4). Improvement of PEF (difference > 0 between PEF obtained at follow-up versus baseline) was observed in 55% of patients at month 10 (+ 0.4 ± 0.2; 0.1–0.7), 33% of patients at month 22 (+ 0.5 ± 0.6; 0.09–0.9) and 30% of patients at month 30 (+ 0.4 ± 0.2; 0.2–0.6). Change in FVC and FEV1 at month 10 positively correlated with the change in HFMSE score at month 10 (rho = 0.70, *p* < 0.05 and rho = 0.70, p < 0.05, respectively). Change in FEV1 showed a positive correlation with the change in RULM at month 10 (rho = 0.78, *p* < 0.01). The change in FVC, FEV and PEF did not correlate with the change in HFMSE and RULM scores in other follow-up timepoints (*p* > 0.05).

**Table 2:** Changes in FVC, FEV1, PEF, HFMSE, RULM, FSS and SF-36 scores of the whole cohort during nusinersen treatment versus baseline

	Month 10	Month 22	Month 30
Δ FVC (mean ± SD, l)	− 0.0 ± 1.0	+ 0.1 ± 0.3	− 0.0 ± 0.3
Δ FEV1 (mean ± SD, l)	− 0.2 ± 0.5	+ 0.3 ± 1.3	− 0.0 ± 0.2
Δ PEF (mean ± SD, l/s)	+ 0.1 ± 0.9	+ 0.3 ± 0.7	+ 0.3 ± 0.6
Δ HFMSE (mean ± SD)	+ 0.3 ± 3.8	+ 0.4 ± 4.3	− 0.2 ± 5.6
Δ RULM (mean ± SD)	+ 1.1 ± 2.7	+ 2.05 ± 2.6	+ 0.2 ± 4.8
Δ FSS (mean ± SD)	+ 0.9 ± 6.8	− 0.0 ± 8.3	+ 3.4 ± 8.3
Δ total SF-36 score (mean ± SD)	− 1.0 ± 13.7	+ 6.5 ± 23.2	− 4.8 ± 15.3

*Δ* difference between the score obtained at one timepoint versus baseline, *SD* standard deviation, *l* liter, *s* seconds, *n* number, *FVC* forced vital capacity, *FEV1* forced expiratory volume in the first second, *PEF* peak expiratory flow, *HFMSE* Hammersmith Functional Motor Scale Expanded, *RULM* Revised Upper Limb Module, *MCID* minimal clinically important difference, *FSS* Fatigue Severity Scale, *SF-36* The 36-Item Short Form Health Survey

### Correlation of pulmonary function with quality of life and fatigue

The change in the total SF-36 score over the whole treatment period did not correlate with the change in FVC, FEV1 and PEF at any timepoint of nusinersen treatment (*p* > 0.05). Change in FEV1 at month 10 positively correlated with the change in RP domain of SF-36 (rho = 0.64, *p* < 0.05), as well as with the change in VT domain (rho = 0.65, *p* < 0.05) and PCS domain of SF-36 10 months after nusinersen initiation (rho = 0.61, *p* < 0.05). Positive correlation was observed between the change in PEF at month 10 and the change in RP domain at month 10 (rho = 0.70, *p* < 0.05). At month 22, the change in FEV1 showed a positive correlation with the change in PF domain of SF-36 (rho = 0.52, *p* < 0.05). At month 30, no correlation between any domain of SF-36 and the change in FVC, FEV1 and PEF was observed (*p* > 0.05).

The change in PEF at month 10 negatively correlated with the change in FSS at month 10 (rho = − 0.70, * p* < 0.05). Negative correlation was observed between the change in PEF at month 30 and the change in FSS at month 10 (rho = − 0.75, *p* < 0.05) and at month 22 (rho = − 0.89, *p* < 0.01).

### Subgroup analysis

Exploratory subgroup analysis according to SMA type (2 vs. 3 and 3a vs. 3b), *SMN2* copy number (< 4 vs. ≥ 4), baseline HFMSE score (< 35 vs. ≥ 35), previous spinal fusion (spondylodesis), use of NIV and use of PEG showed no significant differences in mean change of FVC, FEV1 and PEF at any timepoint during follow-up period. There was a trend toward improvement of mean PEF at month 30 under nusinersen treatment in patients without scoliosis as compared to patients with scoliosis (+ 0.8 ± 0.3 vs. + 0.0 ± 0.5, *p* = 0.053). Ambulatory patients showed statistically significant improvement of mean PEF at month 30 when compared to non-ambulatory patients (+ 0.8 ± 0.5 vs. − 0.0 ± 0.5, *p* < 0.05). SMA patients with presence of fatigue at baseline showed statistically significant improvement of mean PEF at month 10, as compared to SMA patients without fatigue at baseline (+ 0.6 ± 0.9 vs. − 0.4 ± 0.5, *p* < 0.05) (supplementary table 1 and 2). Regarding fatigue, ambulatory and non-ambulatory patients showed no statistically significant difference (*p* > 0.05). Patients with and without fatigue at baseline showed no statistically significant differences in age at therapy start, *SMN2* copy number (< 4 vs. ≥ 4), baseline mean HFMSE score, presence of scoliosis or previous spinal fusion (spondylodesis).

## Discussion

In this study, we analyzed the effect of nusinersen on pulmonary function in adult SMA patients and observed no overall changes during a treatment period of 30 months. Although we observed an improvement in mean PEF at all timepoints and in mean FVC and FEV1 at month 22, the results failed to reach statistical significance. The potential explanation for this finding could be the fact that pulmonary function in SMA patients remains stable in adult age both in early-onset and late-onset SMA [5, 6]. In pediatric SMA patients, Chacko et al. reported that nusinersen slowed down respiratory decline during the first year of treatment [11]. Studies showed benefits of nusinersen in adult SMA patients as well, regarding pulmonary function [12–15]. For example, in a study comprising 19 adult SMA patients, Walter et al. found that peak cough flow significantly improved six months after initiation of nusinersen treatment in adult SMA patients, compared to baseline [12]. An Italian multicentric study (116 included adult patients) observed an increase in FVC but only in ambulatory SMA type 3 patients and an increase in FEV1 in the whole SMA type 3 population 14 months after the initiation of nusinersen treatment [13]. De Wel et al. also reported in a monocentric study that FVC and PEF remained stable over the course of 14 months of nusinersen treatment in 16 adult patients with SMA types 3 and 4, with transient increase in FVC at month 6 [14]. The PEF reflects the strength of expiratory muscles, in absence of bronchial obstruction. For example, Suarez et al. showed that patients with amyotrophic lateral sclerosis and Duchenne muscular dystrophy had a lower PEF than the healthy control group [22]. Maximal expiratory pressure showed improvement after nusinersen treatment in the study of Duong et al. (multicentric study, 42 adult SMA patients) [15]. Future studies in larger SMA cohorts and conducted during longer follow-up periods are needed to conclude if these findings represent normal fluctuations of respiratory function or the effect of nusinersen on pulmonary function as well as to assess the clinical relevance of these results. Lung function seems to be a suitable longitudinal outcome measure until early adulthood [5], but the question is if this also reflects to the adult SMA patients.

Similar to the study of Maggi et al. [13], our ambulatory SMA patients had significant improvement of mean PEF at month 30 after treatment initiation, compared to non-ambulatory patients (+ 0.8 ± 0.5 vs. − 0.0 ± 0.5). SMA patients in our cohort with fatigue at baseline also showed significant improvement of mean PEF early after treatment initiation, when compared to patients without fatigue at baseline (change in mean PEF at month 10: + 0.6 ± 0.9 vs. − 0.4 ± 0.5). Further descriptive statistics revealed that in our cohort patients with presence of fatigue at baseline were less severely affected than patients without fatigue (HFMSE score at baseline: 28.4 ± 22.2 vs. 16.7 ± 23.3), although this did not reach statistical significance (*p* > 0.05), probably due to the small sample size. This is similar to our previous study in which patients with milder disease severity and later disease onset also experienced more fatigue [23]. One can, therefore, assume that the significant improvement in PEF in the subgroup of patients with fatigue is due to better nusinersen response of patients with milder disease severity at baseline, as previously reported [10]. Assessment of pulmonary function during nusinersen therapy might be most important in non-ambulatory patients and patients without fatigue at baseline in everyday practice, as they continue to slightly deteriorate in pulmonary function during nusinersen treatment, while ambulatory patients and patients with fatigue rather show improvement.

Changes in motor and pulmonary function positively correlated with each other at month 10 of nusinersen treatment in our SMA cohort. Similarly, Trucco et al. showed that FVC positively correlated with RULM and HFMSE in a natural history study on respiratory function in patients with SMA type 2 and 3 [6]. This could further strengthen the hypothesis that improvement in the function of respiratory muscles follows improvement in function of peripheral muscles. Furthermore, our study showed that the change in FEV1 and PEF positively correlates with the change in physical domains (RP, RF, VT, PCS) of QoL early after treatment initiation. Also, the change in FSS score negatively correlated with the change in PEF at month 10. These correlations of specific domains of QoL and fatigue with the change in respiratory function during nusinersen treatment have not been assessed by previous studies so far. They indicate that improvements in respiratory function also in mildly affected ambulatory patients are of clinical relevance.

While our study has the advantage of a comparatively long follow-up of an adult SMA patient cohort, the main limitations are the relatively small number of included patients due to monocentric design of the study, absence of control group and loss of follow-up data. Moreover, polysomnography was not performed. Further prospective and multicentric studies in larger numbers of patients are needed to fully assess the impact of nusinersen on pulmonary function in adult SMA and its correlation with QoL and fatigue.

In conclusion, respiratory function remained stable during the long period of nusinersen treatment in most adult patients with SMA in our cohort. Ambulatory patients showed a significant improvement in mean PEF at month 30 after nusinersen initiation, compared to non-ambulatory patients. Pulmonary function improvement was associated with improvement in motor function (according to RULM score), as well as in QoL and fatigue. Pulmonary function tests should be taken into consideration as additional outcome measures, especially in non-ambulatory patients and patients without fatigue at baseline.

## Supplementary Information

Below is the link to the electronic supplementary material.

Supplementary file1 (DOCX 20 KB)

## Data Availability

De-identified data will be shared on reasonable request with any qualifed investigator.

## References

[1] Nance JR (2020). Spinal muscular atrophy. Continuum.

[2] Lefebvre S, Bürglen L, Reboullet S, Clermont O, Burlet P, Viollet L, Benichou B, Cruaud C, Millasseau P, Zeviani M (1995). Identification and characterization of a spinal muscular atrophy-determining gene. Cell.

[3] Lunn MR, Wang CH (2008). Spinal muscular atrophy. Lancet.

[4] Finkel RS, Mercuri E, Meyer OH, Simonds AK, Schroth MK, Graham RJ, Kirschner J, Iannaccone ST, Crawford TO, Woods S, Muntoni F, Wirth B, Montes J, Main M, Mazzone ES, Vitale M, Snyder B, Quijano-Roy S, Bertini E, Davis RH, Qian Y, Sejersen T, Care SMA (2018). Diagnosis and management of spinal muscular atrophy: Part 2: Pulmonary and acute care; medications, supplements and immunizations; other organ systems; and ethics. Neuromuscul Disord.

[5] Wijngaarde CA, Veldhoen ES, van Eijk RPA, Stam M, Otto LAM, Asselman FL, Wösten-van Asperen RM, Hulzebos EHJ, Verweij-van den Oudenrijn LP, Bartels B, Cuppen I, Wadman RI, van den Berg LH, van der Ent CK, van der Pol WL (2020). Natural history of lung function in spinal muscular atrophy. Orphanet J Rare Dis.

[6] Trucco F, Ridout D, Scoto M, Coratti G, Main ML, Muni Lofra R, Mayhew AG, Montes J, Pane M, Sansone V, Albamonte E, D'Amico A, Bertini E, Messina S, Bruno C, Parasuraman D, Childs AM, Gowda V, Willis T, Ong M, Marini-Bettolo C, De Vivo DC, Darras BT, Day J, Kichula EA, Mayer OH, Navas Nazario AA, Finkel RS, Mercuri E, Muntoni F (2021). Respiratory trajectories in type 2 and 3 spinal muscular atrophy in the iSMAC cohort study. Neurology.

[7] Mercuri E, Darras BT, Chiriboga CA, Day JW, Campbell C, Connolly AM, Iannaccone ST, Kirschner J, Kuntz NL, Saito K, Shieh PB, Tulinius M, Mazzone ES, Montes J, Bishop KM, Yang Q, Foster R, Gheuens S, Bennett CF, Farwell W, Schneider E, De Vivo DC, Finkel RS (2018). Nusinersen versus sham control in later-onset spinal muscular atrophy. N Engl J Med.

[8] Finkel RS, Mercuri E, Darras BT, Connolly AM, Kuntz NL, Kirschner J, Chiriboga CA, Saito K, Servais L, Tizzano E, Topaloglu H, Tulinius M, Montes J, Glanzman AM, Bishop K, Zhong ZJ, Gheuens S, Bennett CF, Schneider E, Farwell W, De Vivo DC (2017). Nusinersen versus sham control in infantile-onset spinal muscular atrophy. N Engl J Med.

[9] Darras BT, Chiriboga CA, Iannaccone ST, Swoboda KJ, Montes J, Mignon L, Xia S, Bennett CF, Bishop KM, Shefner JM, Green AM, Sun P, Bhan I, Gheuens S, Schneider E, Farwell W, De Vivo DC (2019). Nusinersen in later-onset spinal muscular atrophy: Long-term results from the phase 1/2 studies. Neurology.

[10] Hagenacker T, Wurster CD, Günther R, Schreiber-Katz O, Osmanovic A, Petri S, Weiler M, Ziegler A, Kuttler J, Koch JC, Schneider I, Wunderlich G, Schloss N, Lehmann HC, Cordts I, Deschauer M, Lingor P, Kamm C, Stolte B, Pietruck L, Totzeck A, Kizina K, Mönninghoff C, von Velsen O, Ose C, Reichmann H, Forsting M, Pechmann A, Kirschner J, Ludolph AC, Hermann A, Kleinschnitz C (2020). Nusinersen in adults with 5q spinal muscular atrophy: a non-interventional, multicentre, observational cohort study. Lancet Neurol.

[11] Chacko A, Sly PD, Ware RS, Begum N, Deegan S, Thomas N, Gauld LM (2022). Effect of nusinersen on respiratory function in paediatric spinal muscular atrophy types 1–3. Thorax.

[12] Walter MC, Wenninger S, Thiele S, Stauber J, Hiebeler M, Greckl E, Stahl K, Pechmann A, Lochmüller H, Kirschner J, Schoser B (2019). Safety and treatment effects of nusinersen in longstanding adult 5q-SMA type 3—a prospective observational study. J Neuromuscul Dis.

[13] Maggi L, Bello L, Bonanno S, Govoni A, Caponnetto C, Passamano L, Grandis M, Trojsi F, Cerri F, Ferraro M, Bozzoni V, Caumo L, Piras R, Tanel R, Saccani E, Meneri M, Vacchiano V, Ricci G, Soraru' G, D'Errico E, Tramacere I, Bortolani S, Pavesi G, Zanin R, Silvestrini M, Politano L, Schenone A, Previtali SC, Berardinelli A, Turri M, Verriello L, Coccia M, Mantegazza R, Liguori R, Filosto M, Marrosu G, Siciliano G, Simone IL, Mongini T, Comi G, Pegoraro E (2020). Nusinersen safety and effects on motor function in adult spinal muscular atrophy type 2 and 3. J Neurol Neurosurg Psychiatry.

[14] De Wel B, Goosens V, Sobota A, Van Camp E, Geukens E, Van Kerschaver G, Jagut M, Claes K, Claeys KG (2021). Nusinersen treatment significantly improves hand grip strength, hand motor function and MRC sum scores in adult patients with spinal muscular atrophy types 3 and 4. J Neurol.

[15] Duong T, Wolford C, McDermott MP, Macpherson CE, Pasternak A, Glanzman AM, Martens WB, Kichula E, Darras BT, De Vivo DC, Zolkipli-Cunningham Z, Finkel RS, Zeineh M, Wintermark M, Sampson J, Hagerman KA, Young SD, Day JW (2021). Nusinersen treatment in adults with spinal muscular atrophy. Neurol Clin Pract.

[16] McDermott MP, Mirek E, Mazzone ES, Main M, Glanzman AM, Duong T, Young SD, Salazar R, Pasternak A (2018). Ambulatory function in spinal muscular atrophy: age-related patterns of progression. PLoS ONE.

[17] Quanjer PH, Tammeling GJ, Cotes JE, Pedersen OF, Peslin R, Yernault JC (1993). Lung volumes and forced ventilatory flows. Report working party standardization of lung function tests, European Community for Steel and Coal. Official statement of the European Respiratory Society. Eur Respir J Suppl.

[18] Mazzone ES, Mayhew A, Montes J, Ramsey D, Fanelli L, Young SD, Salazar R, De Sanctis R, Pasternak A, Glanzman A, Coratti G, Civitello M, Forcina N, Gee R, Duong T, Pane M, Scoto M, Pera MC, Messina S, Tennekoon G, Day JW, Darras BT, De Vivo DC, Finkel R, Muntoni F, Mercuri E (2017). Revised upper limb module for spinal muscular atrophy: development of a new module. Muscle Nerve.

[19] Main M, Kairon H, Mercuri E, Muntoni F (2003). The Hammersmith functional motor scale for children with spinal muscular atrophy: a scale to test ability and monitor progress in children with limited ambulation. Eur J Paediatr Neurol.

[20] Krupp LB, LaRocca NG, Muir-Nash J, Steinberg AD (1989). The fatigue severity scale. Application to patients with multiple sclerosis and systemic lupus erythematosus. Arch Neurol.

[21] SF-36 Health Survey (original version) language recalls. http://www.qualitymetric.com. Accessed Nov 2018

[22] Suárez AA, Pessolano FA, Monteiro SG, Ferreyra G, Capria ME, Mesa L, Dubrovsky A, De Vito EL (2002). Peak flow and peak cough flow in the evaluation of expiratory muscle weakness and bulbar impairment in patients with neuromuscular disease. Am J Phys Med Rehabil.

[23] Binz C, Schreiber-Katz O, Kumpe M, Ranxha G, Siegler H, Wieselmann G, Petri S, Osmanovic A (2021). An observational cohort study on impact, dimensions and outcome of perceived fatigue in adult 5q-spinal muscular atrophy patients receiving nusinersen treatment. J Neurol.

