# Supplementary material for: An observational cohort study on pulmonary function in adult patients with 5q-spinal muscular atrophy under nusinersen therapy
Source: J Neurol. 2023 Apr 16;270(7):3616–22. doi: 10.1007/s00415-023-11711-4 (PMC10106012; doi:10.1007/s00415-023-11711-4)
Supplement: Supplementary file 1 — Supplementary file1 (DOCX 20 KB) [file 415_2023_11711_MOESM1_ESM.docx]

Supplementary table 1. Changes in FVC, FEV1 and PEF in ambulatory vs. non-ambulatory patients

|  | Month 10 | | Month 22 | | Month 30 | |
| --- | --- | --- | --- | --- | --- | --- |
|  | ambulatory | non-ambulatory | ambulatory | non-ambulatory | ambulatory | non-ambulatory |
| Δ FVC (mean ± SD, l) | + 0.0 ± 0.0  (n=5) | - 0.0 ± 0.3  (n=8) | + 0.0 ± 0.3  (n=5) | + 0.1 ± 0.4  (n=3) | + 0.2 ± 0.2  (n=5) | - 0.1 ± 0.3  (n=7) |
| Δ FEV1 (mean ± SD, l) | + 0.2 ± 0.3  (n=4) | - 0.2 ± 0.6  (n=8) | + 0.2 ± 0.3  (n=3) | + 0.9 ± 1.9  (n=4) | - 0.0 ± 0.2  (n=4) | - 0.1 ± 0.2  (n=6) |
| Δ PEF (mean ± SD, l/s) | + 0.2 ± 1.3  (n=4) | + 0.1 ± 0.6  (n=7) | + 0.4 ± 0.9  (n=4) | +0.2 ± 0.4  (n=2) | + 0.8 ± 0.5 *  (n=4) | - 0.0 ± 0.5 *  (n=6) |

N – number of patients; Δ – difference between the score obtained at one timepoint versus baseline; SD – standard deviation; l – liter; s – seconds; FVC – forced vital capacity; FEV1 – forced expiratory volume in the first second; PEF – peak expiratory flow; * - p<0.05

Supplementary table 2. Changes in FVC, FEV1 and PEF in patients with fatigue vs. patients without fatigue

|  | Month 10 | | Month 22 | | Month 30 | |
| --- | --- | --- | --- | --- | --- | --- |
|  | with | without | with | without | with | without |
| Δ FVC (mean ± SD, l) | + 0.0 ± 0.3  (n=6) | - 0.0 ± 0.4  (n=5) | - 0.0 ± 0.3  (n=3) | + 0.0 ± 0.4  (n=3) | + 0.0 ± 0.2  (n=6) | - 0.1 ± 0.1  (n=3) |
| Δ FEV1 (mean ± SD, l) | - 0.2 ± 0.6  (n=7) | - 0.0 ± 0.4  (n=4) | + 0.7 ± 1.6  (n=4) | - 0.3 ± 0.2  (n=2) | + 0.0 ± 0.1  (n=6) | - 0.3 ± 0.1  (n=2) |
| Δ PEF (mean ± SD, l/s) | + 0.6 ± 0.9 *  (n=6) | - 0.4 ± 0.5 *  (n=4) | + 0.5 ± 0.6  (n=3) | + 0.0 ± 1.3  (n=2) | + 0.3 ± 0.7  (n=6) | - 0.0 ± 0.3  (n=2) |

N – number of patients; Δ – difference between the score obtained at one timepoint versus baseline; SD – standard deviation; l – liter; s – seconds; FVC – forced vital capacity; FEV1 – forced expiratory volume in the first second; PEF – peak expiratory flow; * - p<0.05
